# Finding Friends Online: Online Activities by Deaf Students and Their Well-Being

**DOI:** 10.1371/journal.pone.0088351

**Published:** 2014-02-10

**Authors:** Helen Blom, Marc Marschark, Mathijs P. J. Vervloed, Harry Knoors

**Affiliations:** 1 Behavioural Science Institute, Radboud University Nijmegen, Nijmegen, The Netherlands; 2 National Technical Institute for the Deaf, Rochester Institute of Technology, Rochester, New York, United States of America; 3 Royal Dutch Kentalis, Nijmegen, The Netherlands; University of Utah, United States of America

## Abstract

Generally, deaf and hard of hearing (D/HH) children have fewer friends than hearing peers and their friendships are of a lower quality. The research hypothesis was that using the computer to communicate with new online friends through social network sites or playing games with offline friends is associated with D/HH friendship qualities, because it removes certain communication barriers D/HH face in offline communication settings. With online questionnaires the relation between computer use and online, mixed (offline friend who you also speak in online settings), and offline friendship quality of D/HH and hearing students (18–25 years) was compared in both the Netherlands (n = 100) and the United States (n = 122). In addition, the study examined whether the different friendship qualities were related to the participants' well-being. Results showed that, in general, D/HH students' friendship qualities and levels of well-being were similar to their hearing peers. The quality of the mixed friendships was positively related to well-being. Furthermore, the frequency of pc use with both online and offline friends was positively related to friendships qualities in both hearing and D/HH students. A combination of the online and offline friendship seems to be the most important friendship type for both hearing and D/HH students and it is worthwhile to encourage this friendship type.

## Introduction

Deaf and hard-of-hearing (D/HH) adolescents have more difficulties developing and maintaining friendships than hearing adolescents. Online activities have become a natural part of adolescents' lives and online communication could be a more comfortable setting for D/HH adolescents than face-to-face communication. The question now arises whether D/HH adolescents' online activities are valuable for the quality of their friendships and for their well-being.

Compared to their hearing peers D/HH adolescents generally have fewer friends and those friendships are of a lower quality [1]. Essential for adolescents' development, friendships are seen as voluntarily initiating, maintaining and terminating reciprocal relationships. High-quality friendships are related to higher levels of socio-emotional well-being [2], positive social development [3] and better school adjustment [4]. Additionally, adolescents with fewer friends are more likely to drop out of school early, become involved in criminal activities and to develop a psychopathology [5].

Höllinger and Haller [6] compared social networks among different countries and found that on average, Americans between 18 and 24 years of age tend to have more friends than people in European countries. This might be explained by the fact that the definition of a friendship differs between the United States and Europe. In the United States, friends are more widely and casually defined by the activities they share [7]. Americans make a quicker progress towards calling someone a friend than Europeans do. European people hold more gradations in friendships, ranging from casual acquaintances to intimate friends [6]. In contrast to their European counterparts, as fast as the Americans acquire new friends, they are also able to more easily say goodbye to them. Obviously, these differences in friendship definitions might influence perceptions of friendship quality.

### Friendships in deaf and hard of hearing adolescents

Piso, Knoors and Vervloed [8] suggest that the difficulties D/HH children experience in establishing friendships might be due to their geographical distance from other peers, which hinders them “hanging out” with peers. With regard to establishing friendships with hearing peers, communication problems are one of the barriers that deaf adolescents face. Other problems include the challenge to deaf children of engaging in conversations with others, primarily due to misunderstandings and impatience on the part of their hearing conversation partners. Those difficulties are shown to be related to fewer socialization skills in deaf persons [9]. Mainstreamed deaf students specifically have been reported to prefer to socialize with other D/HH children and to lack close friendships within their schools [10]. Furthermore, compared to their hearing peers, deaf persons tend to show poorer mental health: They report more loneliness, a higher risk of psychosocial problems, and a lower general well-being than their hearing peers [11], [12]. With regard to the influence of friendships in D/HH adolescents, Wolters, Knoors, Cillessen and Verhoeven [13] showed that positive relationships of deaf adolescents with their hearing classmates in grades 6 and 7 were related to higher levels of well-being.

### Online communication and social relationships

Nowadays, the Internet has become a natural part of our social lives. Mainly used to communicate with other people, Instant Messaging and Social Network Sites have been accepted as an alternative social environment for people [14]. Although a lot of attention has been paid to studies showing a positive relation between Internet use and loneliness and depression, there are more studies showing that the Internet does not negatively affect existing face-to-face relationships or a person's psychological well-being [14], [15]. Valkenburg and Peter [16] did find that Internet communication was negatively related to personal well-being, overall. However, this relationship changed into a positive one when closeness to a friend was included as a mediator: communication with close friends through the Internet was positively related to adolescents' well-being. In another study on the influence of online communication on adolescents' well-being, Valkenburg and Peter [17] showed that Internet communication is positively related to the time they spend with their friends and the quality of existing friendships and, indirectly, adolescents' well-being. In addition, close relationships can be established and maintained through the Internet, and the breadth, depth and quality of those relationships are highly similar to face-to-face relationships [18]. Chan and Cheng [19] compared the quality of online friendships with the quality of offline friendships and found that the quality of offline friendships was higher than that of online friendships. However, the differences in friendship qualities for relationships that lasted more than one year tended to converge over time.

Of particular interest here is the use of Social Network Sites (SNSs), defined as web-based services that give individuals the opportunity to (1) set up a public profile within a bounded framework, (2) make a list of other users with whom they share a connection and (3) scroll through their list of connections and those made by others within the framework [20]. SNSs are seen as a new means of online communication, with their own idiosyncrasies [21]. Take for example MySpace and Facebook, both seen as friend-networking sites. These websites are used by a large number of adolescents for whom the most common motivations are “to keep in touch with old friends”, “to keep in touch with current friends”, and “to make new friends” [21]. Subrahmanyam, Reich, Waechter and Espinoza [22] studied the activities and motives of young people on SNSs. They found that persons between 18 and 30 years of age used SNSs primarily for social reasons that involved people from their offline lives. The most reported activities on SNSs by the participants suggested that they use SNSs to interconnect with others: most of the time on SNSs was spent on reading and responding to messages and posts on profiles and to browse their friends' pages. A comparable study with high-school students showed the same results in motives and activities on SNSs with a small addition: high school students reported more often to have SNSs to fill up their free time and reported more often to update their status or account. With some SNSs, the primary goal is not to keep in touch with friends, but to provide and seek information through sites such as Twitter or to be entertained, through sites such as YouTube.

### Internet and social life

The development of the Internet has changed the nature of social networks. Where there used to be a time one had only offline friends, networks of online friendships have been developed and evolved to overlap the offline networks [23].

Studies about the influence of computer use on friendship quality are mainly limited to socially anxious adolescents. Desjarlais and Willoughby [24] examined whether the use of a computer with a friend, either in person or online, would be positively related to friendship quality in adolescents with and without social anxiety. The social compensation hypothesis suggests that adolescents with high levels of social anxiety benefit from more computer use with their friends with regard to their friendship quality than adolescents with social anxiety who show less computer use with friends. In contrast, the rich-get-richer theory states that non-anxious adolescents might experience extra benefit from using computers to enrich their friendship quality, while uncomfortable adolescents do not. Regardless of their social anxiety, Desjarlais and Willoughby found a positive relationship between computer use and friendship quality in adolescent girls, supporting both the social compensation theory and the rich-get-richer theory. In boys, social anxiety moderated this relationship: high socially anxious boys who showed a higher use of the computer reported more positive friendship qualities compared to those who reported less computer use with friends.

Although deaf adolescents are not necessarily socially anxious, the question arises whether the use of the computer with a friend, in person or online, can also be beneficial for their friendship qualities, as face-to-face communication is often a challenge for them. For deaf adolescents, the computer and the Internet could be valuable tools extending their social lives, as the Internet provides them the opportunity to communicate with deaf and hearing others in modes others than talking and listening [11]. In addition to that, the Internet is relatively anonymous [14], so deaf adolescents do not necessarily have to reveal their hearing status. Also, this alternative way of communicating relieves them from the stress and psychological uneasiness they generally feel in face-to-face communication with others. Barak and Sadovsky [11] found in their study of the Internet use of deaf adolescents in Israel that they were more motivated to use the Internet than their hearing peers and that their Internet use was more intensive. Although the deaf adolescents generally reported lower of levels of well-being than their hearing peers, those who used the Internet more intensively reported similar well-being levels as hearing adolescents.

The current study aimed to compare the frequencies and motivations of online activities in general, and social exchanges in particular, of D/HH and hearing students in both the Netherlands and the United States, together with the quality of their online, mixed, and offline friendships and its relation to the adolescents' well-being. The moderating effects of age and educational setting on friendship quality were also examined. First, the expectation was that among deaf adolescents, more computer use with a friend, in person or online, would be related to a higher friendship quality with friends they communicate with both in offline and online content, supporting the social compensation theory. Second, it is expected that the hearing students, who tend already to be comfortable in social situations, seek out additional devices to extend their social networks. They might benefit more from computer use with a friend than D/HH peers, which would support the rich-get-richer theory. In addition to those two expectations, it was expected that those effects would be stronger in the Netherlands than in the United States, as friendships in the Netherlands are more based on intimacy and close bonds, while Americans tend to base their friendships on casual shared activities and interests. The link between computer use and the deaf-hearing difference thus may be larger in the Netherlands than in the United States. The final expectation was that the quality of the offline, mixed and online friendships all would be positively related to the adolescents' well-being. Exploratory analyses were done for age and educational setting, but no specific predictions were made for the moderating roles of these variables in friendship quality.

## Methods

### Ethics statement

The study was approved by both the ethical committee of the Faculty of Social Sciences, Radboud University Nijmegen and the Institutional Review Board at Rochester Institute of Technology. Informed consent was obtained through an online informed consent form on the first page of the online survey.

### Participants

Participants were 113 D/HH and 109 hearing students from the Netherlands and the United States (see Table 1). Participants' ages ranged from 18 to 26. Regarding their hearing status, 28% of the students reported being deaf, 23% reported being hard of hearing and 49% described themselves as hearing. Among the D/HH students, 37% used speech to communicate, while 30% used sign language and 33% reported using simultaneous communication or both languages.

**Table 1:** Descriptive statistics of the participants (n = 222).

		NL (n = 100)			US (n = 122)			Total (n = 222)
		D/HH	H	Total	D/HH	H	Total	
Gender	Male	13	10	23 (23%)	26	24	50 (41%)	73 (33%)
	Female	34	43	77 (77%)	40	32	72 (59%)	149 (67%)
Age in	Mean	21,0	21,83	21,42	21,09	20,23	20,7	21,02
years	SD	2,52	1,97	2,28	2,18	1,43	1,92	2,11
School	High school mainstream	4	9	13 (13%)	4	1	5 (4%)	18 (8%)
	High school special	13	-	13 (13%)	1	1	2 (2%)	15 (7%)
	Upper Secondary education	13	4	17 (17%)	-	-	-	17 (8%)
	University/NTID/RIT	17	40	57 (57%)	61	54	115 (94%)	172 (77%)
Hearing	Hearing aid left ear	23	-	23	20	-	20	43
equipment	Hearing aid right ear	20	-	20	22	-	22	42
	CI left ear	7	-	7	18	-	18	25
	CI right ear	7	-	7	17	-	17	24
	None	11	-	11	18	-	18	29

### Measures

Dutch and English versions of the questionnaire had been constructed, enquiring about the participant's online activities, friendship qualities and well-being. Demographic information was also obtained from each participant.

#### Online activities

The online activities of the students were examined by questions based on the questionnaire of Reich, Subrahmanyam, and Espinoza [23], containing items about the frequency and duration of Internet use, whether they have a profile on social networking sites and how often they visit their social networking site (1 =  have it open all the time, 6 =  less than once a week). Further questions were asked about their social networking activities (when you visit your social networking site which 3 of the following activities do you do most often: Edit my profile and update my status, browse my friends' profiles, etc) and their motives for using the profile (Why do you have a profile on MySpace, Facebook, or other similar site: Because all of my friends have accounts, to meet new people and to make new friends, etc). Students who did not have a profile on social networking sites were asked how they felt about not having one (1 =  very cut off from face-to-face friends, 3 =  no effect on face-to-face friends) and whether and how often (1 =  several times a day, 5 =  less than once a week) they visit social networking sites.

#### Friendship qualities

Friendship qualities were measured using the short form of the questionnaire of Parks and Floyd [18], which is an 18-item questionnaire covering 7 factors, constructed by Chan and Cheng [19]. The Cronbach alphas of their study can be found in Table 2. Participants indicated on a 7-point scale to what degree they agreed with the items (1 =  strongly disagree; 7 =  strongly agree). Students were asked whether they have a friend they have met online and with whom they only communicate through social networking sites (i.e. online friend). If they did, they answered questions about the friend's gender, age, hearing status and the quality of this friendship. The same questions were asked about having a friend they have met in a face-to-face setting and with whom they communicate in both face-to-face and online settings (i.e. mixed friend) and about having a friend they have met in a face-to-face setting and with whom they only communicate in face-to-face settings (i.e. offline friend). In order to measure the frequency of computer use with a friend, students indicated on a 4-point scale (0 =  never; 3 =  always) how often they used the computer with an online friend (only online), a mixed friend (both in person and online) and an offline friend (only in person).

**Table 2:** The internal consistency, items and factors measuring friendship quality.

Factor	Item
Interdependence	The two of us depend on each other
α offline = .65	We often influence each other's feelings toward the issues we're dealing with
α online = .63	The two of us have little influence on each other's thoughts (R)
	
Breadth	Our communication is limited to just a few specific topics (R)
α offline = .76	Our communication ranges over a wide variety of topics
α online = .83	
	
Depth	I usually tell this person exactly how I feel
α offline = .65	I would never tell this person anything intimate or personal about myself (R)
α online = .77	
	
Code Change	We have developed the ability to 'read between the lines' of each other's messages to figure out what is really on each other's mind
α offline = .63	The two of us use private signals that communicate in ways outsiders would not understand
α online = .79	We have special nicknames that we just use with each other
	
Understanding	I can accurately predict what this person's attitudes are
α offline = .68	I do not know this person very well (R)
α online = .64	
	
Commitment	This relationship is very important to me
α offline = .73	I would make a great effort to maintain my relationship with this person
α online = .72	I do not expect this relationship to last very long (R)
	
Network Convergence	We have introduced each other to members of each other's circle of friends and family*
α offline = .63	This person and I do not know any of the same people (R)
α online = .61	

*The original item from Parks and Floyd (1996) is: “We have introduced (face-to-face or otherwise) each other to members of each other's circle of friends and family”. To avoid possible confusion regarding ‘online friends’, it was decided to delete ‘face-to-face or otherwise’ from the sentence.

#### Well-being

Well-being was measured with the SatisfactionWith Life Scale [25] and The Loneliness Scale [26]. The SWLS (internal consistency Cronbach α = .87 within their sample) contains five items assessing global life satisfaction (e.g., “In most ways my life is close to my ideal”). Students indicated on a 7-point scale their agreement with each item (1 =  strongly disagree; 7 =  strongly agree). The Loneliness Scale (internal consistency Cronbach α = .70 within their sample) measures overall loneliness by covering two factors: emotional loneliness (e.g., “I experience a general sense of emptiness) and social loneliness (e.g., “There are enough people I feel close to”). Students answered 6 items on 5-point scale to what degree the items applied to them (1 =  Yes!; 5 =  No!).

### Procedure

In the Netherlands, students were actively recruited through invitation letters to special education schools, teacher support organizations and organizations for deaf adolescents and through advertisements on several websites. Dutch students participated by sending an e-mail with their age. In the United States, college students were reached through posters at the campus of Rochester Institute of Technology (RIT) which includes the National Technical Institute for the Deaf (NTID).

Students who signed in received their personal link to the online survey through e-mail. Deaf students of two special schools in the Netherlands filled in a paper version of the questionnaire. In one school, an interpreter was available to provide assistance. The survey took about fifteen minutes to complete.

The database of the research will be made publicly available within the Data Archiving and Networked Services (DANS).

## Results

### Group comparisons

ANOVAs and chi-square tests were conducted to examine differences between the United States and the Netherlands and between D/HH and hearing students in online activities, friendship quality and well-being. Table 3 presents the means and standard deviations of the main variables studied.

**Table 3:** Descriptives of main variables divided by country and hearing status.

	The United States						Netherlands					
	D/HH		H		Total		D/HH		H		Total	
	M	Sd	M	Sd	M	Sd	M	Sd	M	Sd	M	Sd
Days online	6,09	1,72	6,66	1,07	6,35	1,48	6,19	1,23	6,68	,96	6,45	1,11
Minutes online	215,68	146,61	245,89	163,06	229,55	154,47	197,55	174,99	222,17	228,38	210,6	204,39
Numbers of SNS profiles	2,06	1,13	2,19	1,14	2,12	1,13	2,31	1,2	2,0	1,19	2,14	1,20
Frequency visiting SNS sites	2,21	1,15	2,23	1,01	2,22	1,08	2,13	1,12	2,08	,84	2,10	,97
Quality online friendship	4,01	,97	3,46	,95	3,79	,99	3,93	1,68	3,39	1,10	3,66	1,41
Quality mixed friendship _c_ *	4,81	1,18	5,5	1,07	5,14	1,18	5,32	1,11	5,21	,81	5,25	,94
Quality offline friendship	4,61	1,24	4,23	1,12	4,44	1,20	4,46	1,48	4,51	1,03	4,49	1,24
Life satisfaction	23,55	6,92	24,88	7,02	24,16	6,97	23,66	7,59	22,38	6,47	22,98	7,01
Loneliness _a_ *	3,32	1,82	2,43	1,98	2,91	1,94	2,21	1,99	2,08	1,74	2,14	1,86

* *p*<.05; a =  country difference, c =  country_hearing status interaction

#### Online activities

No differences were found between the countries and hearing status on how many days per week the students were online, how many minutes they were online, the number of SNS profiles they have, and the frequency of visiting those SNS sites. In addition, chi-square tests showed no differences in use of instant messaging programs and having a SNS profile. As for the activities of students on social network sites, there was only one significant difference between the United States and the Netherlands: regardless of hearing status, 35% of the Americans reported to write comments on other people's page or wall, while 53% of the Dutch students did this (χ^2^(1) = 5.55, *p*<.05).

#### Motives for having a social network site

Significant differences have been found between the countries and hearing status on various motives for having a profile on social networking sites (Table 4). US students are more likely than Dutch students to have a SNS profile because their friends have accounts. Within the US group, almost all hearing students have a profile for that reason, against more than a half of the D/HH students. In contrast, 30% of the D/HH US students have a SNS profile to meet new people, while only 7% of the hearing Americans mentioned this reason. Staying in touch with friends and family was a reason mentioned significantly more often by the Americans than by the Dutch students overall, and it was also mentioned by more D/HH Dutch than hearing Dutch participants. More hearing Dutch students reported having a SNS profile to read private entries or comment on people's profiles than D/HH Dutch students. Further, more D/HH Americans than D/HH Dutch had a SNS profile to voice their opinions on various topics.

**Table 4:** Percentages of students' motives to have a social network site divided by country and hearing status.

Why do you have a profile on social networking sites?	The United States			Netherlands			Total
	D/HH	H	Total	D/HH	H	Total	
Because all of my friends have accounts _a_ *_,c_ *	61	91	75	49	62	56	66
My friend(s) made it for me	2	11	6	2	4	3	5
To make plans with friends I see often	61	50	56	40	43	42	50
To stay in touch with friends I don't see often	89	88	89	72	81	77	83
To meet new people and to make new friends _c_ *	30	7	20	21	19	20	20
To flirt	5	2	3	2	4	3	3
To share my favorite music and video clips	26	32	29	11	19	15	23
To voice my opinions on various topics (social issues, political issues, current events) _c_ *	36	27	32	19	36	28	30
To stay in touch with relatives and family _a_ *_, b_ *	79	71	75	75	51	62	69
To fill up free time/not be bored	61	61	61	43	53	48	55
To read private entries/to comment on people's profiles _a_ *_, b_ *_, c_ *	27	27	27	30	59	45	35
To explore interests such as music, television shows, etc.	38	36	37	15	25	20	29
Other	8	7	7	19	4	11	9

* *p*<.05; a =  country difference, b =  hearing status difference, c =  country_hearing status interaction

#### Computer use in special context

No differences were found between the countries and hearing status in online computer use with an online friend and computer use (online and offline) with a mixed friend. There was a significant difference between D/HH and hearing students in the degree of computer use with an offline friend, *F*(1, 110)  = 6,48, *p*<.05, 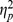  = .06. D/HH students reported to be on a computer together with a friend more frequent than hearing peers (*M* = 2,75, *SD*  =  2,14 and *M* = 1,70, *SD* = 1,31 respectively).

#### Friendship quality

The sample size of each friendship type differs from each other. Whereas the mixed friendship is the most common friendship type (n = 195), the offline friendship follows (n = 113) and the online friendship type is the least frequent (n = 65). No differences were found in the frequency of those friendship types between the countries or hearing status groups. The difference between the quality of online, mixed and offline friendships has been measured with repeated measures ANOVAs. Overall, the quality of the online friendship is significantly lower than the quality of the mixed and offline friendship, *F*(1, 29)  = 28,5, *p*<.001, 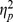  = .5 and *F*(1, 29)  = 7,38, *p*<.05, 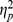  = .2 respectively. The latter two did not differ, *F*(1, 29)  = 4, *p*>.05, 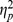  = .12. Within the hearing group, all three groups differed significantly from each other: the quality of the mixed friendship was perceived as highest compared to the online and offline friendship, *F*(1, 11)  = 18,71, *p* = .001, 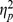  = .63 and *F*(1, 11)  = 8,96, *p*<.05, 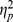  = .45 respectively. The online friendship quality was significantly lower than the offline friendship, *F*(1, 11)  = 8,71, *p*<.05, 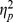  = .44. Within the D/HH group, there was only a significant difference between the online and mixed friendship quality, with the lowest score for the online friendship quality, *F*(1, 17)  = 12,52, *p*<.01, 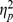  = .42. In both the US and Dutch group, a significant difference has only been found between the online and mixed friendship quality with the latter scoring higher, *F*(1, 19)  = 15,19, *p* = .001, 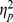  = .44 and *F*(1, 9)  = 13,7, *p*<.01, 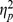  = .6 respectively.

With regard to the difference in mixed friendship quality between both countries and hearing status, there was a significant interaction on the perceived quality of the mixed friendship, *F*(1, 188)  = 5,00, *p*<.05, 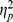  = .03. Hearing students in the US showed higher rates of friendship quality (*M* = 5,5, *SD*  =  1,07) than US D/HH students (*M* = 4,81, *SD*  = 1,18), while no difference in hearing status existed in the Netherlands. Within the D/HH group, it appeared that Dutch students valued the quality of their mixed friendships higher (*M* = 5,32, *SD*  = 1,11) than the D/HH Americans (*M* = 4,81, *SD*  = 1,18). There was no difference between the two countries in the hearing group.

No differences were found for online and offline friendship quality between countries and hearing status.

#### Well-being

There is a significant difference in loneliness between US and Dutch students, *F*(1, 215)  = 5,37, *p*<.05, 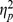 = .02. Regardless of age, gender, school setting and hearing status, US students report more loneliness (*M* = 2,91, *SD*  = 1,94) than Dutch students (*M* = 2,14, *SD*  = 1,85). There were no differences in life satisfaction between the two countries or between the hearing status groups.

### Regressions

Prior to the regression analyses the variables were centered to reduce the multicollinearity between predictor variables. Hierarchical regression analyses were conducted to examine the effect of pc use with a friend, country, hearing status, school setting and age on the online, mixed and offline friendship quality. Each regression analysis was performed separately with online friendship quality, mixed friendship quality and offline friendship quality as the dependent variable. In step 1 of all regression analyses the frequency of pc use with the friend concerned was entered as predictor. In step 2, the variables hearing status, country, school setting and age were entered. In step 3, which was different for each analysis, the interaction terms of the pc use with the control variables from step 2 were entered with the stepwise method to explore their moderating effects. Table 5 provides the results of the significant models of the hierarchical regression analyses.

**Table 5:** Hierarchical regression analyses predicting online, mixed and offline friendship quality from computer use with friend.

		Online friendship quality			Mixed friendship quality			Offline friendship quality	
Predictor		ΔR^2^	β		ΔR^2^	β		ΔR^2^	β
Step 1		.3**			.01			.05*	
Computer use			.54**			.10			.22*
Step 2		.16*			.06*			.07	
Hearing status			−.13			.11		*	.04
Country			.12			.05			.03
School setting			.21			−.12			.16
Age			−.06			−.14			.22*
Step 3		.05			.01			.02	
Computer use x Hearing status			−.01			−.04			.04
Computer use x Country			.23*			−.06			.03
Computer use x Age			.06			−.07			−.07
Computer use x School setting			−.17			.03			−.00
Total *R^2^*	.43**			.06*			.11*		
*N*	64			195			112		

* *p*<.05, ** *p*<.01

The overall model explained 43% of the variance in online friendship quality, *F* (6, 57)  = 7,16, *p*<.001. An interaction effect was found of online pc use with country. In both the US and the Netherlands, there is a positive relation between online pc use and friendship quality (B = .22, SE  = .09, *p*<.05 and B = .55, SE  = .12, *p*<.01 respectively), but this relation appeared to be stronger in the Netherlands. No other significant predictors were found.

The overall model for the prediction of mixed friendship quality showed to be statistically significant with only hearing status, country, school setting and age in it, *F* (5, 189)  = 2,84, *p*<.05, *R^2^* = .07. However, as none of the predictors contribute significantly to mixed friendship quality, the model should be interpreted as nonsignificant. The overall model of offline pc use as a predictor of offline friendship quality was significant with only hearing status, country, school setting and age in it, *F*(5, 106)  = 2,55, *p*<.05. Offline pc use and age were positive predictors of offline friendship quality (*β* = .21, *p*<.05 and *β* = .22, *p*<.05). Regardless of hearing status, playing more computer games together with an offline friend was related to a higher friendship quality. Older students showed a higher offline friendship quality than younger students.

Hierarchical regression analyses were conducted to examine the effects of friendship quality on the adolescents' well-being as a function of country and hearing status. Each regression analysis was performed with both life satisfaction and loneliness as dependent variable. In step 1 of all regression analyses, the friendship variable concerned was entered as predictor. In step 2, the control variables country, hearing status, age and school setting were entered. In step 3, which was different for each analysis, the interaction terms of the friendship quality with country, hearing status, age or school setting were entered with the stepwise method to explore their moderating effects. Table 6 gives the results of the significant hierarchical regression analyses.

**Table 6:** Hierarchical regression analyses predicting well-being from mixed friendship quality and offline friendship quality.

		Mixed friendship quality						Offline friendship quality	
		Life satisfaction			Loneliness			Life satisfaction	
Predictor		ΔR^2^	β		ΔR^2^	β		ΔR^2^	β
Step 1		.04*			.06**			.00	
Friendship quality			.20*			−.25**			.06
Step 2		.04*			.07*			.04	
Hearing status			.01			−.05			.08
Country			−.10			−.17*			−.10
School setting			.11			.06			.22*
Age			−.08			.02			−.09
Step 3		.05**			.03			.15*	
Friendship quality x Hearing status			−.01			.05			.07
Friendship quality x Country			−.04			.07			.4**
Friendship quality x Age			.24*			−.05			−.06
Friendship quality x School setting			−.05			.00			.10
Total *R^2^*	.13**			.11**			.23**		
*N*	195			195			113		

* *p*<.05, ** *p*<.01.

The overall regression models of online friendship quality as a predictor of both Life Satisfaction and Loneliness appeared to be non-significant and could therefore not be interpreted, *F*(5, 59)  = 1,31, *p*>.05 and *F*(5, 59)  = ,34, *p*>.05.

The overall model for the prediction of life satisfaction by mixed friendship quality was significant, *F* (6, 188)  = 4,76, *p*<.001, *R^2^* = .13. There was an interaction effect. Age moderated the association between mixed friendship quality and life satisfaction. Further analyses in which the sample was divided into three age groups, showed that the effect of mixed friendship quality on life satisfaction was only significant for the students in the middle group (mean age  = 21.14, B = 1.67, SE  = .46, *p*<.01) and the older (mean age  = 23,24, B = 3.38, SE  = .74, *p*<.01 respectively), but not for the students who were 1 standard deviation younger than the middle group (mean age  = 19.04). In both significant groups, a positive relation was found between the mixed friendship quality and life satisfaction and this relation was stronger in the oldest group.

The overall model for the prediction of loneliness was significant with only hearing status, country, school setting and age in it, *F*(5, 189)  = 4,49, *p* = .001, *R^2^* = .11. The mixed friendship quality and country were significant predictors of loneliness (*β* = −.23, *p*<.01 and *β* = −.17, *p*<.05 respectively). A higher friendship quality was related to a lower level of loneliness and there was more loneliness in the US than in the Netherlands.

The overall model showed a significant prediction of life satisfaction, *F*(6, 106)  = 5,2, *p*<.01, *R^2^* = .23). There was an interaction effect. Country moderated the association between offline friendship quality and life satisfaction. Further analyses showed that there was a positive association between offline friendship quality and life satisfaction in the Netherlands (B = 3.07, SE  = .79, *p*<.01). A better offline friendship quality was related to a higher life satisfaction among the Dutch participants. A negative relation was found in the US (B = −1.56, SE  = .65, *p*<.05). US participants with higher rates of offline friendship quality showed lower levels of life satisfaction.

The overall model of offline friendship quality as a predictor of loneliness was not significant and could therefore not be interpreted *F*(5, 107)  = 1,42, *p*>.05.

## Discussion

In general, D/HH adolescents were not found to have lower friendship qualities or lower levels of well-being compared to their hearing peers, although previous studies had obtained such findings with younger D/HH students. The quality of their mixed friendships influenced their well-being positively. D/HH adolescents did benefit from computer use with their friends. Those who used the computer with their online and offline friends showed higher friendship qualities.

### Online activities, friendship qualities and well-being

The first aim of this study was to investigate possible differences in online activities, friendship quality and well-being between D/HH and hearing students in the US and the Netherlands.

There were no differences for hearing status or country regarding the frequency of online activities and having a SNS. The motives for using a SNS did differ as a function of hearing status and country. The most important finding was that D/HH Dutch reported to have a SNS to stay in touch with relatives and family more often than hearing Dutch, and US adolescents in general reported this motive more often than Dutch adolescents. The differences can be explained by geographical differences, partly linked with differences in sampling, between both countries: All US participants were university students who were more likely to be away from home and, generally, Americans live further away from each other than Dutch people. Therefore, they are more likely to use SNSs to stay in touch with relatives and family. Compared to the US, the Netherlands is a small country and staying in touch with other people is easier. Where hearing Dutch people can pick up the phone to contact others, deaf people will be more inclined to use SNSs.

More D/HH students than hearing students tend to play computer games and do homework assignments with an offline friend on the computer. Although it hasn't been studied why students choose to do certain activities with each other, communication problems might be a reason for the differences in activities with friends. It is possible that hearing students tend to hang out with their offline friends and do activities outdoors, activities that require dialogues more than computer games do. For D/HH students, sitting at a computer with a friend provides them with a quiet and comfortable environment in which communication costs less effort. This can be a reason why they tend to choose this activity more often than their hearing peers.

In general, the quality of mixed friendships was higher than online and offline friendships. In this study a mixed friendship was defined as having a friend with whom one communicates in both face-to-face situations and through SNSs. This combination of communication settings seems to be beneficial for relationship quality. Previous research also showed that online communication with existing friends is positively related to the quality of the friendship with them [27], [28]. There are several possible explanations for this finding. Vitak sees SNSs as perfect tools to stay in touch with friends and to maintain those relationships, while Valkenburg and Peter state that online communication, in addition to face-to-face contact, stimulates intimate online self-disclosure toward friends which, in turn, influences the friendship quality positively. The combination of face-to-face and online contact appears to result in a better friendship quality than offline or online friendships alone. Alternatively, given the correlational nature of the relevant analyses, the reverse effect might be plausible (if less likely, intuitively): good friendship quality could lead to friends being more likely to meet in both online and face-to-face settings while lower quality friendships could lead to meeting one another only in online or offline settings. The quality of mixed friendships was the only type that differed significantly depending on hearing status and country. Although in general D/HH students do not have mixed friendships with a lower quality than hearing peers, D/HH Americans do. This difference in mixed friendship quality between American D/HH and hearing students was not found in the Dutch group. Although, sampling differences between the US and Dutch participants (i.e. there were no high school students in the US sample) might partly explain this result, it stil is remarkable, as previous Dutch studies showed that D/HH students do have a lower friendship quality than hearing students [1], [8]. The most noticeable difference between the three studies is the age difference. This study consisted of university students, while Piso, Knoors and Vervloed studied high school students and Kouwenberg studied students from both primary and secondary schools. Maybe age plays a role in how adolescents value their friendships. Early adolescence does involve the appreciation of having friends and developing a social status within the classroom [13] and it is possible that D/HH early adolescents are more insecure about their existing friendships than D/HH late adolescents are. This could negatively influence the perception of the quality of the friendships. Furthermore, there was a difference in the friendship type studied. Whereas Piso, Knoors and Vervloed and Kouwenberg studied friendships in general, the current study did take the online aspect into account. It might be that the mix of online and offline communication is the factor that D/HH students don't have a lower friendship quality than hearing students. Further studies should examine the whole age-range from early adolescence to adulthood and take different friendship types into account.

Another finding in the current research was that D/HH Americans had a lower mixed friendship quality than D/HH Dutch students. As noted earlier, this result might be explained by the difference in definition of friendships between the two countries, sampling differences, or students' proximity to their families. Results regarding well-being showed no difference due to hearing status. This is a remarkable finding, as D/HH students were not less satisfied or lonely than hearing peers. This is in contrast with previous findings that showed lower levels of well-being in D/HH persons compared to hearing persons [29], [30]. However, those studies involved students between 4 to 19 years of age, while the current study involved older students. As already stated above, it is possible that younger children who are D/HH are more insecure about their lives than older D/HH children. Future studies about D/HH children's well-being should take the age range into account as well as the issue of whether students are living close to home.

### Computer use and friendship quality

The second aim of the study was to examine whether frequency of computer use with a friend was related to higher online, mixed and offline friendship quality.

Regardless of hearing status, more computer use with an online friend was related to a higher friendship quality. This result is not striking, as the computer is needed to have online contact with friends. But the reverse can be true as well: close friends tend to keep in touch by phone, the Internet, or whatever means available, explaining the more frequent use of the computer by close friends. That the effect between computer use and online friendship quality was stronger for the Netherlands could be the result of the above mentioned difference in the definition of friendships between the two countries. Nevertheless, we cannot rule out the possibility that the American students had most of their friends nearby because most of them lived in dormitories, and they did not need the computer to stay in touch. The finding that frequency of computer use with an online friend is stronger for Dutch persons than for Americans can be explained [7], following the line of reasoning that intimate self-disclosure is easier in online settings than offline settings [31] and that self-disclosure is important for the friendship quality [32],

More computer use with an offline friend was related to a higher friendship quality in both D/HH and hearing students. This result was not in accordance with Mathur and Berndt [33], who studied the relation between the frequency and importance of friends' activities and friendship quality in fourth- and eighth graders. They found that socializing was the most frequent and important activity for those students and that it was positively related to intimacy and prosocial interactions with friends. However, the frequency of media use with friends (watching TV, playing a video game, going to movies, etc.) has only been reported to be related to feelings of inequality within the friendship and not to feelings of intimacy and prosocial interactions. Mathur and Berndt suggested that those feelings are the result of competition within media activities and ownership of the media being used. When media use was reported to be important for the students, a positive relation between intimacy and prosocial interactions was found. On the contrary, sports, play and games appeared to be less frequent and important and not related to friendship quality at all. This suggests that it is not just spending time together that is related to the friendship quality, but it is the type of activity that matters.

In short, the results support the social compensation theory and not the rich-get-richer theory, as D/HH students who use the computer with a friend reported a higher online and offline friendship quality than D/HH students, who do this less. No support for the rich-get-richer theory was found, as hearing students did not benefit more from computer use with friends than the D/HH students. The expectation that the effects of computer use on friendship quality would be stronger in the Netherlands than in the United States was only found for online friendships.

### Friendship quality and well-being

The third aim of the study concerned the relation between online, mixed and offline friendship quality and well-being.

Regardless of hearing status and country, greater mixed friendship quality was associated with less loneliness and more life satisfaction. These findings are in accordance with previous studies on the relationship of general friendship quality with well-being [34], [2]. Positive friendship qualities work as a buffer against negative life events and contribute to higher levels of self-esteem and confidence in the student. At the same time: students who are more satisfied with their lives invest more in their friendships and develop a higher friendship quality than students who are less satisfied with their lives. The relationship of mixed friendship quality and life satisfaction was moderated by age: the effect was only visible in students who were 21 years or older. Although this research has not studied the duration of friendships, it is possible that friendship length is a determining factor in the relation between mixed friendship quality and life satisfaction. Antheunis, Valkenburg and Peter [35] previously found a positive relation between friendship duration and quality and that could affect the life satisfaction.

Offline friendship quality was related to life satisfaction in both countries. The relationship was positive in the Netherlands meaning that higher offline friendship quality was associated with higher life satisfaction. This was in accordance with a previous study [36] that showed that companionship and self-validation were the most important aspects of friendship quality that accounted for the person's happiness. However, a reverse result was found in the US: higher offline friendship quality was associated with less life satisfaction. It is possible that the quantity of the friendship is a factor in the relation between friendship quality and life satisfaction. Powdthavee [37] studied the degree of life satisfaction in relation to interaction with friends and found that a person who spends more time with their friends valued his life as higher than someone who had fewer interactions with his friends. As the US students within this study were all living away from their friends at home, they spend less time with those friends and this has a negative association with both their life satisfaction and friendship qualities. Further studies should take into account the time students are able to spend with their friends.

### Limitations and implications

This study had some limitations. First, was the fact that all US participants lived away from their parents, whereas the Dutch participants were more likely to live at their parents' houses. The geographical difference could have been a factor in the frequency of several online activities and motivations to be active on SNSs. In addition, the participants in the Netherlands were reached through invitation letters through schools and organizations and online advertisements, while the students in the US were only reached by posters around the campus. This could have affected the composition of the sample group. Second, the nature of the online survey should be considered. The answers cannot be controlled, so it would be ideal to have a combination of an online and offline part in a follow-up study. Furthermore, this study showed the relation between the online, mixed, and offline friendship quality and well-being. How fluctuations in those friendship types affects a person's well-being is unknown. Future longitudinal studies should investigate the influence of those qualities over time.

A positive finding in this study was that compared to hearing peers, D/HH students do not have a lower friendship quality and well-being. It showed that D/HH students are not experiencing more difficulties with starting and maintaining friendships than hearing adolescents do and that they are not more lonely or less satisfied with their lives.

Within this study it became apparent that, for both hearing and D/HH students, there were no harmful effects from online friendships on well-being. It seems that having an online friend through SNSs doesn't necessarily relate to higher levels of loneliness. The Internet can be used by D/HH children who feel more comfortable in online settings to connect with other peers and to develop friendships.

A combination of the online and offline friendship seems to be the most important friendship type for both hearing and D/HH students. It has a positive relation with well-being. Encouragement of these mixed friendships seems worthwhile.
